# Evidence of Chinese Herbal Medicine Use From an Economic Perspective: A Systematic Review of Pharmacoeconomics Studies Over Two Decades

**DOI:** 10.3389/fphar.2022.765226

**Published:** 2022-05-05

**Authors:** Xiaomo Xiong, Xiangxiang Jiang, Gang Lv, Jing Yuan, Minghui Li, Z. Kevin Lu

**Affiliations:** ^1^ Department of Clinical Pharmacy and Outcomes Sciences, University of South Carolina, Columbia, SC, United States; ^2^ School of Public Health, Nanjing Medical University, Nanjing, China; ^3^ Department of General Surgery, The First Medical Center of PLA General Hospital, Beijing, China; ^4^ Department of Pharmacy Administration, Fudan University, Shanghai, China; ^5^ Department of Clinical Pharmacy and Translational Science, University of Tennessee Health Science Center, Memphis, TN, United States

**Keywords:** economic evaluation, Chinese herbal medicines, traditional Chinese medicine, systematic review, pharmacoeconomics

## Abstract

**Objectives:** Pharmacoeconomics evaluation (PE) is increasingly used in the healthcare decision-making process in China. Little is known about PE conducted in Chinese Herbal Medicines (CHMs). We aimed to systematically review trends, characteristics, and quality of PE of CHMS.

**Methods:** We systematically searched both Chinese (CNKI, WanFang, and VIP) and English (Pubmed) databases. Studies were included if they were PE studies comparing both costs and outcomes between two or more interventions published in Chinese or English. Assessment of the quality of studies was conducted using the Quality of Health Economic Analyses (QHES) instrument. *T*-test and Chi-square tests were used to compare the studies before and after the first edition of *China Guidelines for PE* published in 2011, and between studies published in Chinese and English.

**Results:** A total of 201 articles were included. There was an increasing trend of PE studies on CHMs during the study period. The top three studied diseases were central nervous system (CNS), mental, and behavioral disorders; cardiovascular diseases; and blood, immune and endocrine diseases. The average QHES score for the included studies was 63.37. Cost-effectiveness analysis (CEA) accounted for the majority (76.6%) of the included studies. Only a quarter of the articles (27.4%) were funded, and there were significantly more studies funded after the publication of *China guidelines for PE*. About 96.5% of studies did not specify evaluation perspectives and 89.6% of studies had a sample size of less than 300. Around half of the studies (55%) used incremental analysis, but only a few of them considered using a threshold. Half of the studies lacked sensitivity analysis. There was no significant improvement in the quality of studies published after the publication of *China Guidelines for PE*, and English articles had significantly higher quality than Chinese articles.

**Conclusion:** This study identified several problems in PE studies on CHMs, including having small sample sizes, lacking necessary research elements, and using single evaluation methods. The quality of PE studies on CHMs was not sufficient. Researchers need to understand the standardized way to conduct PE studies and improve the quality and level of PE studies on CHMs.

## Background

Pharmacoeconomics evaluation (PE) refers to a branch of health economics that compares costs and health outcomes of pharmaceuticals and related intervention (Reeder, 1995; Drummond and McGuire, 2001; Stahl, 2008; Rawlins et al., 2010). PE research aims to form an optimal scheme for decision-making and improve the overall efficiency of medical resource allocation (Drummond and McGuire, 2001; Miller, 2005; Rawlins et al., 2010). The PE is applied in many decision-making practices, including guiding clinical use of drugs, pricing innovative drugs, and making health insurance formularies (Miller, 2005; Clement et al., 2009; Rawlins et al., 2010; Ngorsuraches et al., 2012; Dakin et al., 2015; Jönsson, 2015). Due to the development of evidence-based healthcare and the needs of decision-makers, PE has been developing quickly in China (Li et al., 2009; Yue et al., 2021). After the *China Guidelines for PE* was published in 2011, the number of publications related to PE has been increasing gradually (Liu, 2011; Butt et al., 2019; Yue et al., 2021). Specifically, PE studies in China mainly focus on the comparison among western medicines (87.9%), followed by the comparison between Chinese Herbal Medicines (CHMs) and western medicines (7.0%) and the comparison among CHMs (3.5%) (Li et al., 2001).

CHM is a medicine applied under the guidance of the theory of traditional Chinese medicine, which medicine made of plants, animals, and minerals. CHM has a wide range of meanings and is mainly divided into Chinese medicinal materials and Chinese medicinal preparations, including traditional clinical preparations (including pills, powders, pastes, dan, soup, etc.), Chinese patent medicines, and Chinese medicine granules for compatibility. Our study was limited to pharmacoeconomic evaluations on Chinese herbal medicine in China, so we can exclude other traditional medicines and compare study characteristics before and after the China PE guideline. CHMs have a history of thousands of years and have been being broadly used in clinical practice in China and several other Asian countries, such as Korea and Japan (Pan et al., 2014; Yu et al., 2017). In October 2013, WHO issued the *WHO Traditional Medicine Strategy 2014–2023* to provide guidance to the Member States in managing priorities, regulations, and governance in the area of traditional medicine within their jurisdictions (World Health Organization, 2013). In addition to updating the previous *WHO Traditional Medicine Strategy 2002–2005*, this strategy also aimed to respond to the *World Health Assembly resolution on Traditional Medicine WHA62.13* that urged the integration of traditional medicine into the national health care system (World Health Organization, 2002; World Health Organization, 2013). As one of the few countries that have already integrated traditional medicine into the national healthcare system, CHMs are used as the primary treatment for a variety of diseases in China. Currently, CHMs account for more than 40% of China’s pharmaceutical market (Traditional Chinese Medicine, 2021).

Among the wide variety of CHMs, there are many medications with similar indications (Jiang, 2005; Feng et al., 2006). In such a large number of CHMs, it is critical to make decisions rationally and economically to enhance the use of CHMs and the allocation of limited health care resources. In China’s latest national health insurance list that used PE evidence as one of the selection criteria, CHMs accounted for nearly 50% of the medications listed (National Administration of Traditional Chinese Medicine, 2019). However, there are currently no specific PE guidelines for CHMs or traditional medicine. The trend, characteristics, and quality of PE studies of CHMs remain unknown. To fill the gap in the literature, we aimed to systematically review the trend, characteristics, and quality of the PE studies of CHMs based on Preferred Reporting Items for Systematic Reviews and Meta-Analyses (PRISMA) guidelines. The results of this review would be helpful in informing the use of PE evidence for policymakers in making decisions about CHMs and providing directions on how to promote the quality of PE of CHMs for researchers.

## Methods

### Search Strategy

The search strategy was established based on the population, interventions, comparators, outcomes and study designs (PICOS) and PRISMA guidelines. Specifically, the population was patients involving with CHM treatments, the interventions were treatments including at least one Chinese herbal medicine, the comparators were non-placebo treatments, outcomes were economic health outcomes, and study designs included cost-minimization analysis, cost-benefit analysis, cost-effectiveness analysis, cost-consequence analysis, and cost-utility analysis. We systematically searched both Chinese (CNKI, Wanfang, and VIP) and English (Pubmed) databases from database creation to 31 July 2020. Two categories of search terms were used, including 1) pharmacoeconomics, economic evaluation, cost-benefit analysis, cost-effectiveness analysis, cost-utility analysis, cost-minimization analysis, or cost-consequence analysis; and 2) traditional Chinese medicine, TCMs, Chinese herbal medicines, traditional medicine, Chinese medicine, or herbal medicines. Detailed search strategies are shown in Supplementary Table S1.

### Inclusion and Exclusion Criteria

Articles were included if they were PE studies comparing both costs and outcomes between two or more interventions in Chinese or English. Articles were excluded if they were reviews, letters, comments, and not available for access to the full article.

### Data Extraction

Full articles were retrieved after reading abstracts of records searched. The information extracted from articles included title, the year of publication, disease and/or condition evaluated, types of evaluation methods, funding, first author affiliation, study perspectives, the source of effectiveness, sample size, time horizon, threshold consideration, cost sources identification, interpretation of results, sensitivity analysis, adverse event, and incremental analysis. The disease and/or condition evaluated were classified using Classification of Diseases Tenth Revision (ICD-10) code groupings (World Health Organization, 1992). Data were extracted by two independent reviewers. A third individual was consulted when agreements were not able to be reached.

### Evaluation of Quality of Included Studies

Assessment of the quality of studies was conducted using the Quality of Health Economic Analyses (QHES) instrument, which is a widely used evaluation instrument for the quality of research on health economics (Ofman et al., 2003). The QHES contains 16 items for evaluation in the form of “yes or no” questions selected by eight experts in health economics (Ofman et al., 2003). Each item of the QHES has a weighted point ranged from 1 to 9. If the article failed to meet the requirement of one item, the score of the corresponding item would be counted as 0 point, while if the article met the requirement, then it would be counted as a full score (Ofman et al., 2003). The QHES instrument has a minimum score of 0 and a maximum score of 100 (Ofman et al., 2003).

### Statistical Analysis

The trend of the publication of PE studies on CHMs was plotted based on the number of the publication of each year. In addition, we compared the characteristics and quality of the included studies before and after the publication of *China PE guidelines* in 2011, and between articles published in Chinese and English. The student’s *t*-test was used to compare continuous variables, while Chi-square and Fisher exact tests were used for categorical variables. All analyses were conducted using SAS 9.4 (The SAS Institute, Cary, NC).

## Results

**FIGURE 1:**
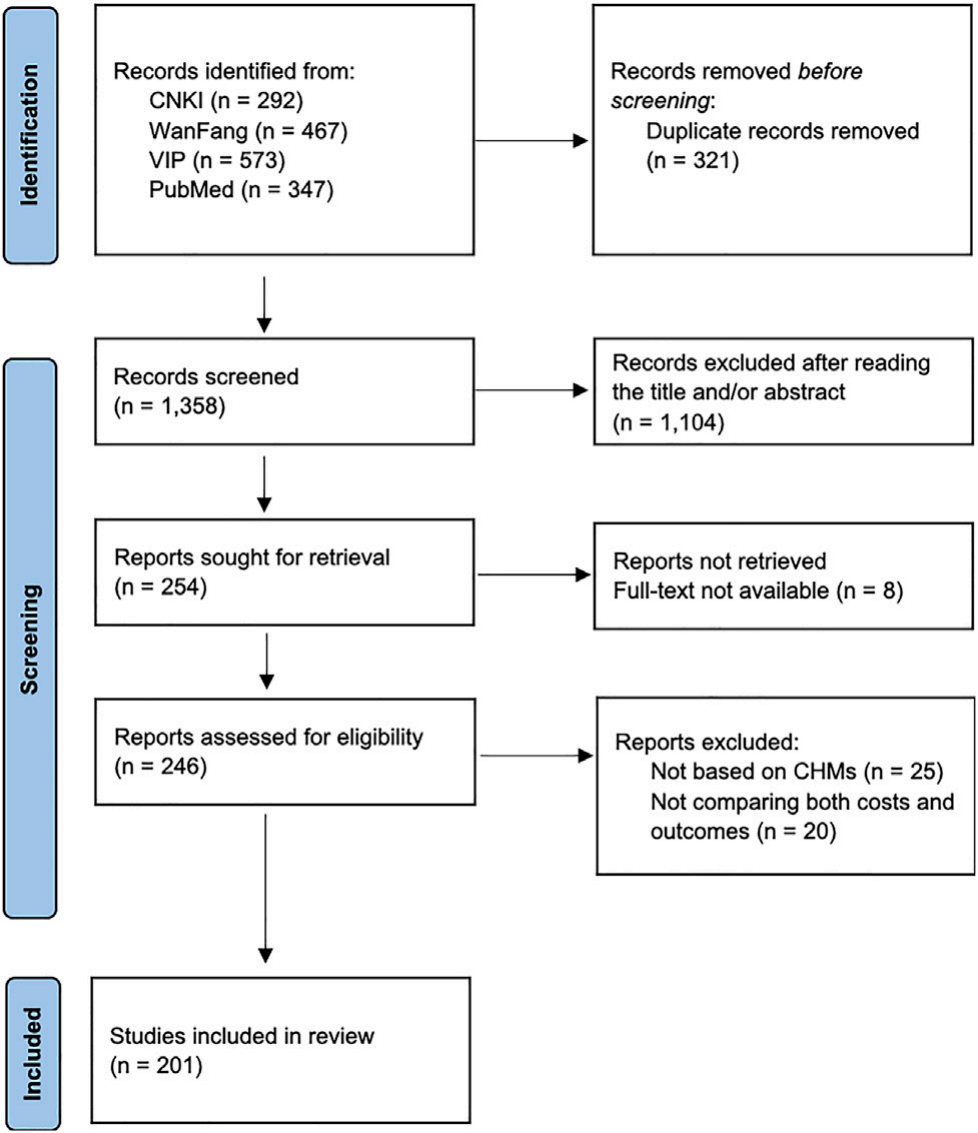
PRISMA flow diagram of the literature screening process. PRISMA, Preferred Reporting Items for Systematic Reviews and Meta-Analyses.

The PRISMA flow diagram of the literature screening process was summarized in Figure 1. A total of 1,679 records were identified after searching the databases. After removing duplicates, 1,358 records were screened. Based on inclusion and exclusion criteria, 1,104 articles were excluded after reading titles and abstracts. During the full-text review, 53 articles were excluded and 201 articles were included. Specifically, among the included articles, 196 studies (97.5%) were published in Chinese and eight studies (2.5%) were published in English. The information on the year, authors and title of the included studies is shown in Supplementary Tables S2, S3.

**FIGURE 2:**
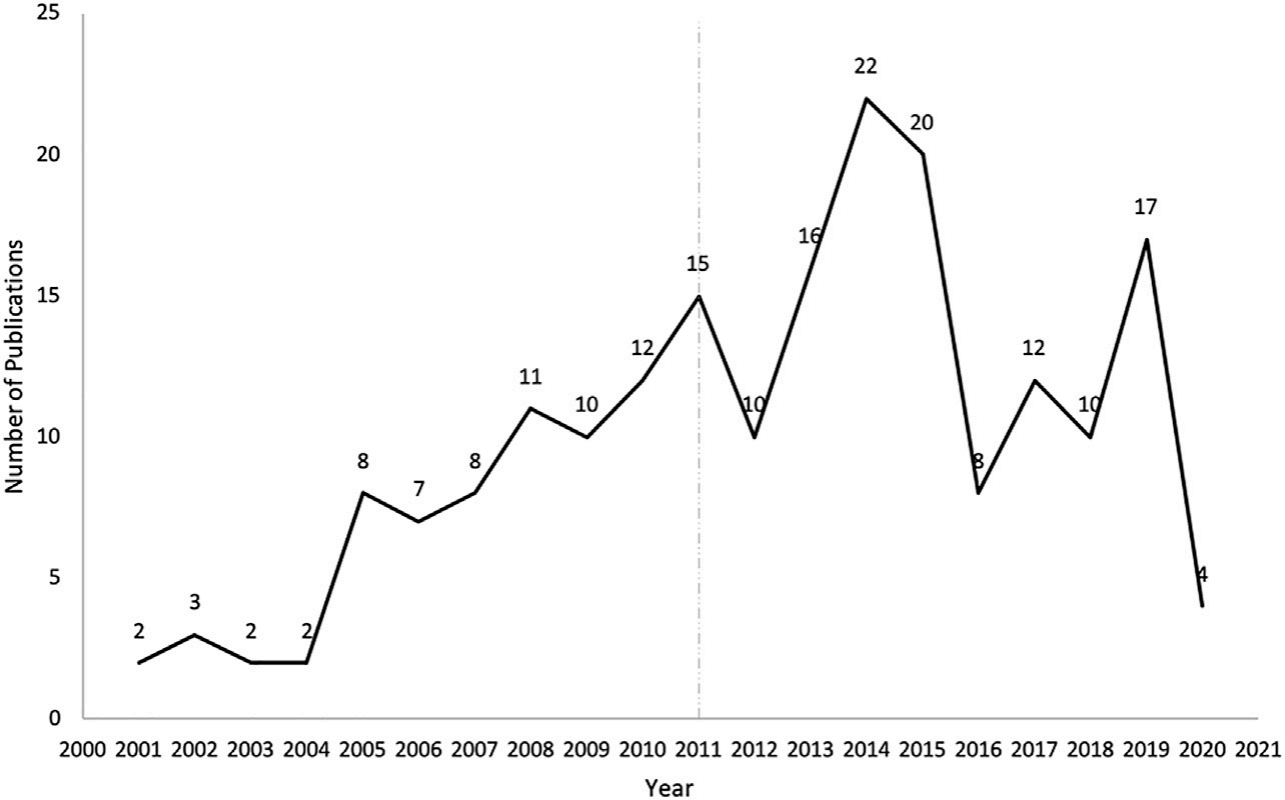
The number of publications of pharmacoeconomics evaluation of herbal medicine by years. The vertical line for 2011 represents the publication of China’s PE guidelines.

**FIGURE 3:**
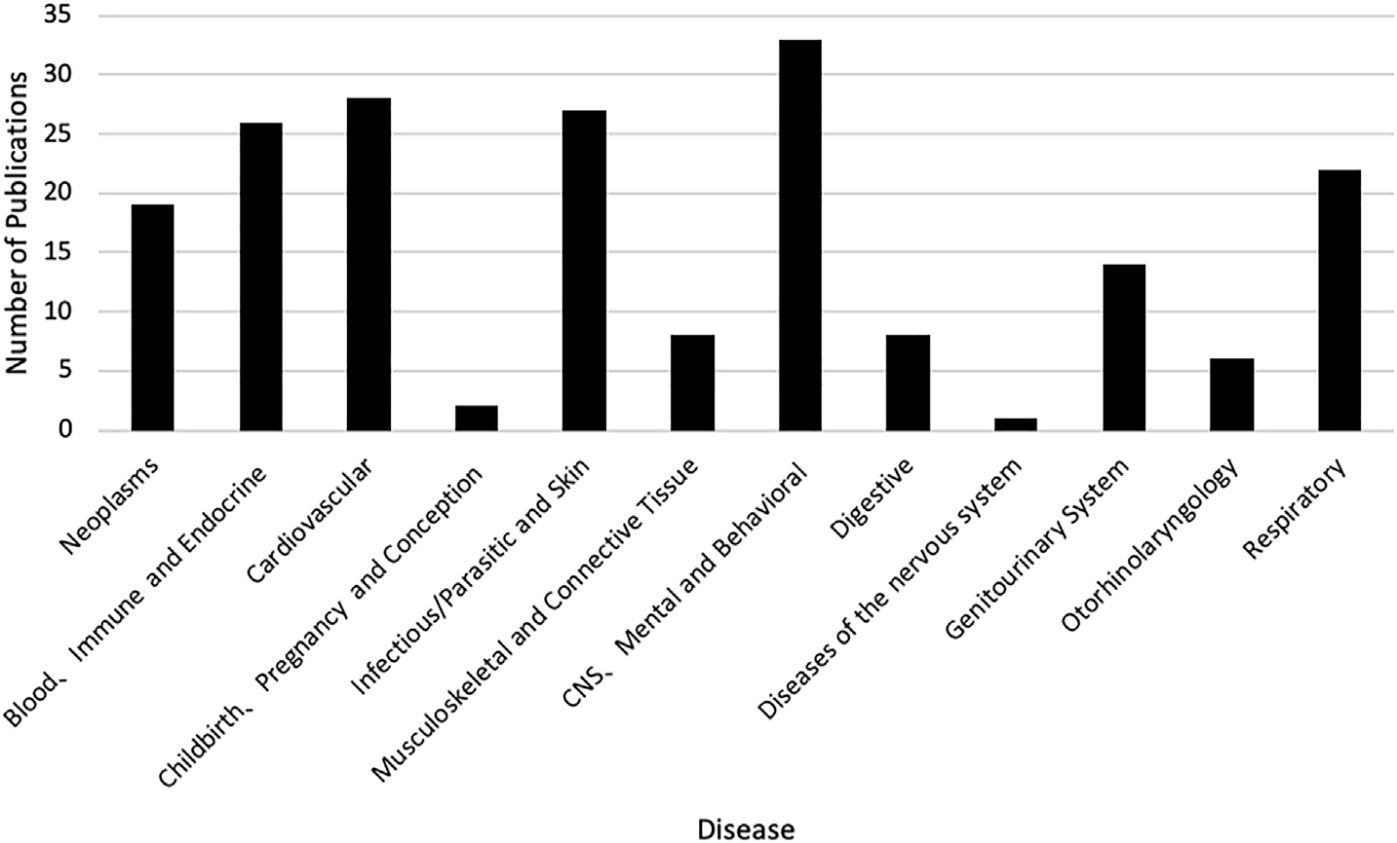
The number of publications of pharmacoeconomics evaluation of herbal medicine by diseases. CNS, Central nervous system.

There was an increasing trend of PE studies of CHMs from 2001 to 2020, which peaked in 2014 (Figure 2). The top three studied diseases were central nervous system (CNS), mental, and behavioral disorders; cardiovascular diseases; and blood, immune and endocrine diseases (Figure 3). For the treatment group, 145 articles used CHM alone and 56 used CHM in combination with western medicine. For the control group, 75 studies used CHM alone, 28 used CHM in combination with western medicine, and 98 used western medicine combination therapy.

**FIGURE 4:**
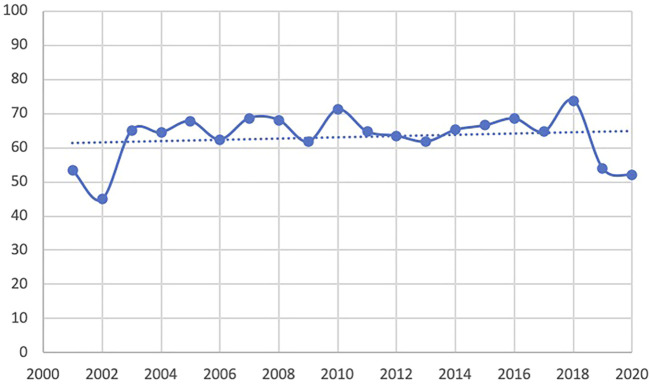
QHES scores by published year. QHES, Quality of Health Economic Studies.

The trend line of the QHES shows that the quality of the PE studies in CHM improve slightly the past two decades overall. However, the quality appears to have dropped in 2019 and 2020 (Figure 4). The average QHES score of the included studies was 63.37. CEA studies accounted for most of the published studies (87.5%), and the majority of the affiliation of the first author was hospital institutions (73.1%), followed by academic institutions (25.4%) and industries (1.5%). Only around one-fourths (27.4%) of PE studies on CHMs reported funding sources, and the majority of studies did not specify evaluation perspectives (96.5%). In terms of the source of effectiveness, observational studies accounted for 33.8% of the PE studies, while clinical trials and literature review accounted for 59.7% and 6.5%, respectively. More than three-fourths of the included PE studies (76.6%) had a sample size of less than 300, and 10 articles (5.0%) did not report sample size. A total of 146 studies (72.6%) had a relatively short study period (≤24 months), and 20 articles did not report a time horizon (10.0%). The majority of studies (86.1%) reported its cost source identification. In terms of incremental analysis, 120 articles conducted incremental analysis, which accounted for 40.3%. However, only four articles (2.0%) considered a threshold that is necessary for incremental analysis. More than 90% of studies have interpreted the results. More than half of the studies (50.7%) did not consider adverse events, and 28 articles (13.9%) did not conduct cost identification. 80 articles (39.8%) did not conduct sensitivity analysis to control for uncertainty (Table 1). Based on Table 2, in general, the main issues with the PE studies on CHM are the lack of study perspective, the lack of description of study period/time horizon and discounting, and the unclear expression of models, assumptions, limitations, and biases. On the other hand, most of the studies did well in the methodological statement, the expression of health outcomes, and the transparent descriptions of analysis. After the publication of China’s PE guidelines, the descriptions of perspective and research bias in these studies have been enhanced.

**TABLE 1:** Comparison of characteristics of the included studies before and after the publication of the China PE guideline.

	Total *N* = 201, No., mean (%, SD)	Published before the guidelines *N* = 81, No., mean (%, SD)	Published after the guidelines *N* = 120, No., mean (%, SD)	*p*-value	Chinese articles *N* = 193, No., mean (%, SD)	English articles *N* = 8, No., mean (%, SD)	*p*-value
QHES scores	63.37 (12.17)	64.85 (11.22)	62.37 (12.67)	0.156	62.71 (11.62)	79.25 (14.18)	<0.001
Types of evaluation methods				0.730			0.001
CEA	176 (87.6)	70 (86.4)	106 (88.3)		172 (89.1)	4 (50.0)	
CBA	4 (2.0)	2 (2.5)	2 (1.7)		4 (2.1)	0 (0.0)	
CUA	6 (3.0)	2 (2.5)	4 (3.3)		3 (1.6)	3 (37.5)	
Comprehensive analysis	11 (5.5)	4 (4.9)	7 (5.8)		11 (5.7)	0 (0.0)	
Others	4 (2.0)	3 (3.7)	1 (0.8)		3 (1.6)	1 (12.5)	
Treatment group				0.074			0.854
CHM alone	145 (72.1)	64 (79.0)	81 (67.5)		139 (72.0)	6 (75.0)	
CHM in combination with western medicine	56 (27.9)	17 (21.0)	39 (32.5)		54 (28.0)	2 (25.0)	
Control group				0.909			0.268
CHM alone	75 (37.3)	29 (35.8)	46 (38.3)		74 (38.3)	1 (12.5)	
CHM in combination with western medicine	28 (13.9)	11 (13.6)	17 (14.2)		28 (14.5)	1 (12.5)	
Western medicine alone	98 (48.8)	41 (50.6)	57 (47.5)		91 (47.2)	6 (75.0)	
First author affiliation				0.489			0.017
Hospital institutions	147 (73.1)	63 (77.8)	84 (70.0)		142 (73.6)	5 (62.5)	
Industry	3 (1.5)	1 (1.2)	2 (1.7)		3 (1.6)	0 (0.0)	
Academic institution	51 (25.4)	17 (21.0)	34 (28.3)		48 (24.9)	3 (37.5)	
Funding				0.054			0.001
Yes	55 (27.4)	16 (19.8)	39 (32.5)		49 (25.4)	6 (75.0)	
No	146 (72.6)	65 (80.2)	81 (67.5)		144 (74.6)	2 (25.0)	
Study perspectives				0.245			<0.001
Yes	7 (3.5)	1 (1.2)	6 (5.0)		1 (0.5)	6 (75.0)	
No	194 (96.5)	80 (98.8)	114 (95.0)		192 (99.5)	2 (25.0)	
Source of effectiveness				0.003			<0.001
Clinical trial	120 (59.7)	59 (72.8)	61 (50.8)		114 (59.1)	6 (75.0)	
Observational studies	68 (33.8)	17 (19.8)	51 (42.5)		66 (34.2)	2 (25.0)	
Literature review	13 (6.5)	5 (6.2)	8 (6.7)		13 (6.7)	0 (0.0)	
Sample size				0.274			<0.001
≤100	64 (31.8)	28 (34.6)	36 (30.0)		62 (32.1)	2 (25.0)	
101–300	90 (44.8)	38 (46.9)	52 (43.3)		89 (46.1)	1 (12.5)	
301–1,000	26 (12.9)	10 (12.3)	16 (13.3)		23 (11.9)	3 (37.5)	
>1,000	11 (5.5)	1 (1.2)	10 (8.3)		9 (4.7)	2 (25.0)	
N/A	10 (5.0)	4 (4.9)	6 (5.0)		10 (5.2)	0 (0.0)	
Time horizon				0.056			<0.001
≤1 month	19 (9.5)	14 (17.3)	5 (4.2)		19 (9.8)	0 (0.0)	
1–6 months	33 (16.4)	13 (16.0)	20 (16.7)		33 (17.1)	0 (0.0)	
7–12 months	43 (21.4)	17 (21.0)	26 (21.7)		42 (21.8)	1 (12.5)	
13–24 months	51 (25.4)	17 (21.0)	34 (28.3)		51 (26.4)	0 (0.0)	
>24 months	35 (17.4)	12 (14.8)	23 (19.2)		28 (14.0)	7 (87.5)	
N/A	20 (10.0)	8 (9.9)	12 (10.0)		20 (10.9)	0 (0.0)	
Cost source identification				0.173			0.801
Yes	173 (86.1)	73 (90.1)	100 (83.3)		166 (86.0)	7 (87.5)	
No	28 (13.9)	8 (9.9)	20 (16.7)		27 (14.0)	1 (12.5)	
Incremental analysis				0.098			0.375
Yes	120 (59.7)	54 (66.7)	66 (55.0)		114 (59.1)	6 (75.0)	
No	81 (40.3)	27 (33.3)	54 (45.0)		79 (40.9)	2 (25.0)	
Threshold consideration				0.477			<0.001
Yes	4 (2.0)	1 (1.2)	3 (2.5)		1 (0.5)	3 (37.5)	
No	197 (98.0)	80 (98.8)	117 (97.5)		192 (99.5)	5 (62.5)	
Result interpretation and recommendation				0.458			0.746
Yes	177 (88.1)	73 (90.1)	104 (86.7)		169 (87.6)	8 (100.0)	
No	24 (11.9)	8 (9.9)	16 (13.3)		24 (12.4)	0 (0.0)	
Adverse event consideration				0.405			0.861
Yes	99 (49.3)	37 (45.7)	62 (51.7)		95 (49.2)	4 (50.0)	
No	102 (50.7)	44 (54.3)	58 (48.3)		98 (50.8)	4 (50.0)	
Sensitivity analysis				0.213			0.015
Yes	121 (60.2)	53 (65.4)	68 (56.7)		113 (58.5)	8 (100.0)	
No	80 (39.8)	28 (34.6)	52 (43.3)		80 (41.5)	0 (0.0)	

QHES, Quality of Health Economic Studies; CEA, Cost-effectiveness analysis; CBA, Cost-benefit analysis; CUA, Cost-utility analysis; N/A, Not applicable.

**TABLE 2:** Comparison of QHES of the Included Studies before and after the Publication of the China PE guideline.

QHES items	Total *N* = 201, No. (%)	Published before the guidelines *N* = 81, No. (%)	Published after the guidelines *N* = 120, No. (%)	*p*-value
QHES-1: Objective	129 (64.2)	50 (61.7)	79 (65.8)	0.552
QHES-2: Perspective	6 (3.0)	0 (0.0)	6 (5.0)	0.041
QHES-3: Best available source for variable estimates	121 (60.2)	60 (74.1)	61 (50.8)	0.001
QHES-4: Subgroup analysis described	186 (92.5)	75 (92.6)	111 (92.5)	0.980
QHES-5: Uncertainty handled	122 (60.7)	53 (65.4)	69 (57.5)	0.259
QHES-6: Incremental analysis	118 (58.7)	53 (65.4)	65 (54.2)	0.112
QHES-7: Methodology stated	199 (99.0)	81 (100.0)	118 (98.3)	0.243
QHES-8: Time horizon and discount	7 (3.5)	2 (2.5)	5 (4.2)	0.520
QHES-9: Appropriate measurement of costs	179 (89.1)	73 (90.1)	106 (88.3)	0.690
QHES-10: Primary outcome stated	201 (100.0)	81 (100.0)	120 (100.0)	1.000
QHES-11: Valid and reliable health outcomes measures	201 (100.0)	81 (100.0)	120 (100.0)	1.000
QHES-12: Transparent descriptions of analysis	196 (97.0)	80 (98.8)	115 (95.8)	0.231
QHES-13: Statement and justification of models, assumptions, and limitations	50 (24.9)	15 (18.5)	35 (29.2)	0.087
QHES-14: Bias	57 (28.4)	16 (19.8)	41 (34.2)	0.026
QHES-15: Accurate conclusions and recommendations of the study	198 (98.5)	81 (100.0)	117 (97.5)	0.152

In Table 1, compared to studies published before the publication of *China PE guideline*, the QHES score was not significantly different from the research published afterward (*p* = 0.156). In addition, except for the source of effectiveness (*p* = 0.003), there was no significant difference between other characteristics between studies before and after the guideline was published (*p* > 0.05). Table 1 shows the difference between studies published in Chinese and English. Compared to the studies published in Chinese, research published in English has a significantly higher quality (79.25 vs. 62.71, *p* < 0.001). Moreover, compared to studies published in Chinese, those published in English were more likely to use CUA (*p* = 0.001), be conducted by academic institutions (*p* = 0.017), be funded (*p* = 0.001), report study perspectives (*p* < 0.001), use clinical trials as the source of effectiveness (*p* < 0.001), have a larger sample size (*p* < 0.001), longer time horizon (*p* < 0.001), and use threshold to compare the cost-effectiveness and sensitivity analysis to control for uncertainty (*p* = 0.015).

## Discussion

In this review, we found that the overall trend of PE studies on CHMs was increasing from 2001 through 2020, and reached the peak in 2014. Considering there were more studies funded after the *China guidelines for PE* were published, the guidelines might play a certain role to stimulate the studies of PE, and the decline since 2014 might be due to a lack of specific PE guidelines for CHMs. However, only a quarter of PE studies on CHMs were funded. The support from funding sources, such as government and academic institutions, might be still not enough. Therefore, related government agencies might consider increasing the financial support for PE studies on CHMs. At the same time, other funding sources, such as academic institutions and insurance payers, might need to be encouraged to actively participate in the research regarding PE on CHMs. The top three diseases studied in the included articles were chronic diseases, including CNS, mental, and behavioral disorders; cardiovascular diseases; and blood, immune and endocrine diseases. This is consistent with clinical practice, where CHMs are often used to control and delay the progression of chronic diseases (Chan et al., 2010; Layne and Ferro, 2017).

We also found that the evaluation methods mainly focused on CEA. CEA studies of CHMs accounted for the majority of the published studies. One of the disadvantages when using CEA is that it is difficult to compare between groups when different health output indicators are used in two comparison groups (Stahl, 2008). Researchers should choose appropriate evaluation methods according to the characteristics of intervention measures, the availability of data, and the objectives and requirements of evaluation, rather than using CEA alone (Jakubiak-Lasocka and Jakubczyk, 2014; Muennig and Bounthavong, 2016). In addition, outcome indicators were also single among PE studies of CHMs, which might lead to neglect of other effects of target drugs. Therefore, evaluation methods and outcome indicators should be diversified when conducting in future PE studies on CHMs.

We also assessed the quality of PE studies on CHMs using the QHES instrument in this review and found that the quality was not high. Therefore, improving the quality of research is one of the most urgent actions to enhance the validity of PE studies of CHMs. There were several methodological problems in PE studies on CHMs. First, the research perspective affects the scope and estimation of cost, and the selection and calculation of effect indicators and the consistency of the research perspective must be maintained in the same study. However, the majority of the research did not mention the research perspective. Study perspectives play an important role in the evaluation of PE. The study perspective needs to be determined first, and then a series of evaluation processes such as study design, analysis methods, cost, and effectiveness measurement can be measured and determined (Sanders et al., 2016). Cost components and estimates differ greatly from one study perspective to another (Sanders et al., 2016). Therefore, researchers should specify the study perspective when to conduct the evaluation of PE on CHMs. Second, the sample size was small for most of the studies, which might lead to sufficient study power to compare the effectiveness and costs between the study and control groups (Dupont and Plummer, 1990). Third, nearly half of the articles did not use incremental analysis, and almost all of the articles did not consider a threshold to compare the cost and effectiveness. Many articles only reported costs and effectiveness directly, which was difficult to provide useful information for decision-makers. In future research, more in-depth analysis is needed. Finally, many studies did not use sensitivity analysis. Sensitivity analysis is a necessary method to ensure the external validity of the results (Claxton, 2008), because the cost in the base-case analysis is usually from a single source but different provinces in China may have different prices and costs for CHMs. In addition, we found that the majority of PE studies (97.5%) included were published in Chinese. This may be because CHM is mainly used in China, so most of the articles are published in China for clinical reference. However, in our review, we found that the quality of the PE studies of CHM published in English is higher than those in Chinese. Therefore, researchers should be encouraged to publish their research results in journals in English, which may improve the overall quality of PE studies on CHM. On the other hand, considering that more than 50% of treatments in China involve the CHM, journals in English may also consider including more PE studies on CHM to improve the dissemination value of relevant literature.

By comparing the studies before and after the publication of *China guidelines for PE*, we found that there was no significant improvement in the quality of studies published after the publication of the guideline. Taking into account the difference between CHMs and western medicines, especially the difference in clinical use, specific guidelines for PE on CHMs might be needed to guide the application of PE for CHMs (Chan et al., 2010). In addition, the affiliation of the first author was mostly from the hospital, and although they are experts in clinical practice, they might do not have a thorough understanding of health economics and PE, which might lead to methodological shortcomings and biases in interpreting results. Therefore, it is necessary to establish the PE guidelines and norms in line with the characteristics of CHMs, and define the research path, so as to provide references for future research and improve the quality of the research on PE studies on CHMs. We also compared studies published in Chinese and English, the quality of English articles is significantly higher compared to articles in Chinese journals. This might be due to the higher submission standards and the higher standards of reviewers. Specific quality differences can also be reflected in methodology. All of the studies published in English used sensitivity analysis, which could increase the robustness of the results. At the same time, there were more articles reporting study perspectives and using larger sample size and longer time horizon.

This study has several limitations. First, the information we extracted was based on the PE guideline of western medicine, which might not be applicable to the evaluation of PE studies on CHMs. However, there is no instrument specifically for assessing the quality of the PE of CHMs. Therefore, the QHES is the best evaluation tool we could choose. Second, quality evaluation was based on subjective judgments, which might be biased. However, the quality evaluation process was conducted by two reviewers, which could reduce the bias. Third, due to the large body of literature included in our study, we did not review the difference in different diseases, while we categorized the diseases using ICD codes to a disease type. Further studies may be needed to identify the difference in using pharmacoeconomics for different diseases specifically.

## Conclusion

According to our review, there are several methodological problems in PE studies on CHMs, including single evaluation methods, lack of study perspectives, small sample size, lack of incremental analysis, and lack of sensitivity analysis. Based on the QHES score, the quality of PE studies on CHMs was not high. This might be due to the lack of fudging, and specific PE guidelines for CHMs. Therefore, related government agencies and other funding sources might consider increasing the financial support of PE studies of CHMs. Meanwhile, specific PE guidelines for CHMs are needed to improve the application of PE of CHMs.

## Data Availability

The original contributions presented in the study are included in the article/Supplementary Material, further inquiries can be directed to the corresponding author.
