# Supplementary material for: Evidence of Chinese Herbal Medicine Use From an Economic Perspective: A Systematic Review of Pharmacoeconomics Studies Over Two Decades
Source: Front Pharmacol. 2022 May 5;13:765226. doi: 10.3389/fphar.2022.765226 (PMC9117622; doi:10.3389/fphar.2022.765226)
Supplement: Supplementary file 1 [file Table1.DOCX]

**Table S1. Search strategies**

| Data sources | Search strategies | |
| --- | --- | --- |
| PubMed | #7 | #5 AND #6 |
|  | #6 | #3 OR #4 |
|  | #5 | #1 OR #2 |
|  | #4 | (((traditional Chinese medicine[MeSH Terms]) OR (traditional medicine[MeSH Terms])) OR (Chinese medicine[MeSH Terms])) OR (herbal medicine[MeSH Terms]) |
|  | #3 | (((traditional Chinese medicine[Title/Abstract]) OR (traditional medicine[Title/Abstract])) OR (Chinese medicine[Title/Abstract])) OR (herbal medicine[Title/Abstract]) |
|  | #2 | (((((((cost-effectiveness analysis[MeSH Terms]) OR (cost-utility analysis[MeSH Terms])) OR (cost-benefit analysis[MeSH Terms])) OR (cost-consequence analysis[MeSH Terms])) OR (cost-minimization analysis[MeSH Terms])) OR (pharmacoeconomics[MeSH Terms])) OR (economic evaluation[MeSH Terms])) OR (economic analysis[MeSH Terms]) |
|  | #1 | (((((((cost-effectiveness analysis[Title/Abstract]) OR (cost-utility analysis[Title/Abstract])) OR (cost-benefit analysis[Title/Abstract])) OR (cost-consequence analysis[Title/Abstract])) OR (cost-minimization analysis[Title/Abstract])) OR (pharmacoeconomics[Title/Abstract])) OR (economic evaluation[Title/Abstract])) OR (economic analysis[Title/Abstract]) |
| CNKI | (SU = '药物经济学' OR SU = '成本-效果分析' OR SU = '成本-效益分析' OR SU = '成本-效用分析' OR SU = '最小成本分析') AND (SU = '中药' OR SU = '中医' OR SU = '传统医疗' OR SU = '传统药物' OR SU = '天然药物' OR SU = '中成药' OR SU = '中西药') | |
| WanFang | (主题：“药物经济学” + 主题：“成本-效果分析” + 主题：“成本-效益分析” + 主题：“成本-效用分析” + 主题：“最小成本分析”) * (主题：“中药” + 主题：“中医” + 主题：“传统医疗” + 主题：“传统药物” + 主题：“中成药” + 主题：“中西药” + 主题：“中西医”) | |
| VIP | (M = 药物经济学 OR M = 成本-效果分析 OR M = 成本-效益分析 OR M = 成本-效用分析 OR M = 最小成本分析) AND (M = 中药 OR M = 中医 OR M = 传统医疗 OR M = 传统药物 OR M = 天然药物 OR M = 中成药 OR M = 中西药) | |

**Table S2. Included Chinese Studies**

| **Number** | **Year** | **Author** | **Title** |
| --- | --- | --- | --- |
| 1 | 2001 | 谢川黔; 叶云; 戚明双 | 独一味片等三种止痛药的费用-效果分析 |
| 2 | 2001 | 王悦; 李鲁; 何炳荣; 张融碧 | 中西降压药的成本—效果分析综合评价 |
| 3 | 2002 | 陈长发 | 中药熏洗与复方氯唑沙宗治疗上、下肢骨关节损伤的费用-效果分析 |
| 4 | 2002 | 谢朝晖; 刘华; 蒋立东 | 三种方法治疗呼吸道感染的费用—效果分析 |
| 5 | 2003 | 王慧明; 曾幼莲 | 4种中成药治疗乳腺增生病的成本-效果分析 |
| 6 | 2003 | 宋招弟 | 乳腺增生病治疗的药物经济学分析 |
| 7 | 2003 | 杨建文; 石凤鸣 | 中药颗粒剂的成本-效益分析 |
| 8 | 2004 | 杨理会 | 4种中药注射液治疗病毒性心肌炎的成本-效果分析 |
| 9 | 2004 | 席薇 | 急性脑梗塞药物治疗方案的成本效果分析 |
| 10 | 2005 | 徐运安 | 中西药不同方案治疗带状疱疹的成本-效果分析 |
| 11 | 2005 | 陶建青; 尹雪冰; 梁佳; 曾强; 朱澜; 康红; 业明 | 3种中成药治疗失眠症的成本-效果分析 |
| 12 | 2005 | 李淑敏 | 中西药3种方案治疗突发性耳聋的成本-效果分析 |
| 13 | 2005 | 周岱翰; 林丽珠; 周宜强; 罗荣城; 刘魁凤; 贾英杰; 陈继跃; 牛喜伟; 苏碧茹; 韩慧; 鲁江; 王树堂 | 非小细胞肺癌三种治疗方案的成本-效果分析 |
| 14 | 2005 | 易学东; 陈科 | 参苓白术散汤剂与单味颗粒剂提高脾阴虚型厌食症患者机体素质的成本-效果分析 |
| 15 | 2005 | 哈力哈娜胡宝荣 | 3种中西药物治疗老年功能性便秘的成本-效果分析 |
| 16 | 2005 | 顾秀琰杨小源张琳 | 3种中药注射剂治疗小儿外感发热的成本-效果分析 |
| 17 | 2005 | 许萍 | 中药消痤汤和维胺酯胶囊治疗寻常性痤疮的药物经济学分析 |
| 18 | 2006 | 费炳红 | 中西药联用治疗泌尿道感染的成本-效果分析 |
| 19 | 2006 | 徐航; 方芸; 黄莉莉 | 中西药2种药物治疗方案治疗前列腺增生症的最小成本分析 |
| 20 | 2006 | 杜书章; 岳晓红 | 稳定型心绞痛三种中西药物治疗方案的成本-效果分析 |
| 21 | 2006 | 余学庆; 李建生; 李素云; 王大中; 王明航 | 两种慢性阻塞性肺疾病治疗方案的成本-效果分析 |
| 22 | 2006 | 路静华 | 中药灌肠法佐治婴幼儿腹泻的成本-效果分析 |
| 23 | 2006 | 王昕 | 脑脉泰胶囊治疗血管性痴呆的随机对照试验及成本效果分析 |
| 24 | 2007 | 李娜 | 治疗小儿急性上呼吸道感染三种用药方案的药物经济学分析 |
| 25 | 2007 | 毛敏 | 2型糖尿病早期中西医结合治疗的成本-效果分析 |
| 26 | 2007 | 毛敏; 余江毅 | 六味地黄软胶囊和银杏叶片对早期2型糖尿病生存质量的影响及成本-效果分析 |
| 27 | 2007 | 马为; 窦增新 | 中西医治疗老年人肺炎治疗方案的成本——效果分析 |
| 28 | 2007 | 戚春梅; 苏文晓; 黄思毅 | 2种治疗用药方案治疗慢性盆腔炎的成本与效果分析 |
| 29 | 2007 | 王宁 | 阿德福韦酯与中药制剂治疗HBeAg阴性慢性乙型肝炎成本—效果分析 |
| 30 | 2007 | 崔丽 | 三种方案治疗急性上呼吸道感染的药物经济学评价 |
| 31 | 2008 | 黄敏; 张相年 | 两种抗肿瘤中成药在中晚期胃癌化疗中应用的药物经济学分析 |
| 32 | 2008 | 谈瑄忠; 石红乔 | 不同用药方案治疗带状疱疹的成本-效果分析 |
| 33 | 2008 | 张建国; 周晓明 | 3种中成药用于治疗心绞痛的成本-效果分析 |
| 34 | 2008 | 郭钻; 陈卫明 | 3种中成药治疗良性前列腺增生症成本-效果分析 |
| 35 | 2008 | 孟凡珍; 王凡; 刘琦; 陈慧肃 | 3种治疗脑梗塞中药的成本-效果分析 |
| 36 | 2008 | 柯炳峰 | 四种治疗病毒性感冒方案的成本-效果分析 |
| 37 | 2008 | 刘敏; 年士恒 | 五种蜜炙中药的药物经济学应用研究 |
| 38 | 2008 | 李红星 | 经皮给予中药辅助治疗小儿病毒性肺炎风热闭肺型的成本-效果分析 |
| 39 | 2008 | 魏长华 | 小儿呼吸道合胞病毒肺炎中西医随机对照治疗方案临床经济学研究 |
| 40 | 2008 | 王阶; 何庆勇; 邢雁伟; 李海霞; 王师菡; 房玉涛 | 基于中西医结合治疗方案的冠心病心绞痛的卫生经济学研究 |
| 41 | 2008 | 何春霞 | 非小细胞肺癌住院病例的成本-效果回顾性研究 |
| 42 | 2008 | 左志燕 | 3种中药注射剂治疗外感发热的成本-效果分析 |
| 43 | 2008 | 陈双英; 伍良知; 文继平; 何晓勤 | 四种中药制剂治疗脑梗死成本-效果分析 |
| 44 | 2008 | 黄宏兴; 邓崇礼; 李颖; 梁祖建; 万雷 | 中药骨康治疗绝经后骨质疏松症卫生经济学分析 |
| 45 | 2009 | 刘壬通 | 两种治疗子宫肌瘤中成药的药物经济学分析 |
| 46 | 2009 | 苏琼华 | 2种中成药治疗方案用于寻常性痤疮的成本-效果分析 |
| 47 | 2009 | 杨楠; 郑利群 | 中西医结合卒中单元治疗缺血性脑卒中的成本-效果分析 |
| 48 | 2009 | 周昕; 李智成; 阙华发; 谢瑞芳; 顾希钧; 刘晓鸫 | 中西医治疗丹毒成本-效果比较分析 |
| 49 | 2009 | 乔萍; 宋惠丽; 王元松 | 2型糖尿病口服降糖药物治疗的药物经济学评价 |
| 50 | 2009 | 黎东生; 杨洁 | 治疗失眠症三种用药方案的成本-效果分析 |
| 51 | 2009 | 王芳 | 黑地黄丸治疗慢性肾衰脾肾两虚证的临床疗效及成本效果分析 |
| 52 | 2009 | 吴洪文; 陈少卿; 肖萍 | 3种治疗乳腺小叶增生中药的成本-效果分析 |
| 53 | 2009 | 曾忠荣; 李日莲 | 八种方案治疗霉菌性阴道炎的成本效果分析 |
| 54 | 2010 | 沈华林; 周婕 | 3种中成药治疗方案用于乳腺增生的成本-效果分析 |
| 55 | 2010 | 屠国昌 | 4种中成药治疗肝纤维化的成本-效果分析 |
| 56 | 2010 | 屠国昌; 吕聪燕 | 4种方案治疗支气管炎的成本-效果分析 |
| 57 | 2010 | 张凤芹; 曲雷鸣; 龚伟 | 中西药联用治疗高脂蛋白血症药物经济学评价 |
| 58 | 2010 | 潘颖超 | 慢性阻塞性肺疾病稳定期辨证治疗的疗效评价与成本-效果分析 |
| 59 | 2010 | 张德珍 | 中药注射剂治疗急性脑梗死的药物经济学分析 |
| 60 | 2010 | 马瑛; 靳学婷 | 6种中药制剂治疗急性脑梗死的成本-效果分析 |
| 61 | 2010 | 张凡珍 | 黑地黄丸治疗慢性肾衰竭的成本—效用分析 |
| 62 | 2010 | 熊一岚; 万力生 | 3种方案治疗儿童原发性遗尿症的药物经济学分析 |
| 63 | 2010 | 诸林俏; 周璇; 黄晨 | 3种中药注射剂治疗不稳定型心绞痛的药物经济学评价 |
| 64 | 2010 | 汪红兵; 张声生; 李振华; 唐旭东; 王新月; 张福文; 陈明; 陶琳; 唐博祥; 朱培一; 吴兵; 李保双; 宋秀江; 刘敏; 查波 | 中医药辨证治疗腹泻型肠易激综合征卫生经济学评价 |
| 65 | 2011 | 颜刚; 李水云 | 3种中成药治疗乳腺小叶增生的成本-效果分析 |
| 66 | 2011 | 路红显 | 354例晚期乳腺癌患者不同疗法的药物经济学分析 |
| 67 | 2011 | 马妍妍; 哈娜 | 中西药2种方案治疗慢性心力衰竭的成本-效果分析 |
| 68 | 2011 | 吴玉波; 王伟巍; 李岩; 张卓伯; 蒋爱华; 马丽颖; 徐艳丽; 孙向菊; 闫虹 | 3种治疗急性缺血性脑卒中药的最小成本分析 |
| 69 | 2011 | 李小东; 秦明珠; 邵志伟 | 中西医2种方案治疗寻常型银屑病的经济学评价 |
| 70 | 2011 | 刘瑜新; 宋晓勇; 张世清; 张红霞; 刘强; 杨磊; 张永州 | 3种用药方案治疗慢性宫颈炎的药物经济学分析 |
| 71 | 2011 | 岑艳华; 陈丽 | 3种方案治疗急性泌尿系统感染的药物经济学分析 |
| 72 | 2011 | 周华; 严鸿兴 | 中西医治疗血虚风燥型手足癣成本—效果分析 |
| 73 | 2011 | 王明学; 陈亚妮 | 两种中医药方法促进胃癌术后肠功能恢复的经济学评价 |
| 74 | 2011 | 夏猛; 覃振林; 唐红珍 | 调理脾胃法治疗抑郁症的卫生经济学评价 |
| 75 | 2011 | 黄剑林 | 两种治疗冠心病中成药的药物经济学分析 |
| 76 | 2011 | 吴宏赟; 胡志强; 吕娟 | 慢性紧张型头痛风瘀候综合治疗方案卫生经济学评价 |
| 77 | 2011 | 李晓菊; 毕雪艳; 姜艳 | 2种中药注射液治疗小儿急性上呼吸道感染的疗效观察与成本-效果分析 |
| 78 | 2011 | 徐珏 | 不同方法治疗2型糖尿病的成本效益分析 |
| 79 | 2011 | 梁盈军; 舒洁倩 | 良附丸等6首方剂中药配方颗粒汤剂与中药饮片汤剂临床治疗的成本－效果分析 |
| 80 | 2011 | 赵志刚 | 中西医结合治疗湿热痹阻型类风湿关节炎的疗效及成本分析 |
| 81 | 2012 | 李文华; 朱文婷; 韩蕾; 高明; 许梦; 赵晶; 李霞; 祁麟; 高成哲; 周晓辉 | 两种中西医结合方案治疗下尿路感染的药物经济学分析 |
| 82 | 2012 | 梁玉屏 | 子宫肌瘤的两种治疗方案药物经济学分析 |
| 83 | 2012 | 魏传良; 王宗丽 | 生津止渴丸联合降糖药用于Ⅱ型糖尿病的疗效及药物经济学评价 |
| 84 | 2012 | 吴声振; 冯惠玲; 雷凯君 | 3种方案治疗痰热型慢性咽炎的疗效及成本分析 |
| 85 | 2012 | 吴飞跃 | 4种中药感冒药的药效与经济学分析 |
| 86 | 2012 | 王诺; 张占军; 常冬 | 运用Markov模型对中药干预的成本-效果分析 |
| 87 | 2012 | 唐毅 | 运用成本—效果分析方法对COPD稳定期优选治疗方案的研究 |
| 88 | 2012 | 于福文; 阎姝 | 3种中药注射剂治疗冠心病心绞痛的成本-效果分析 |
| 89 | 2012 | 刘海青 | 2种方案治疗小儿病毒性肺炎的卫生经济学分析 |
| 90 | 2013 | 杨晨 | 老年男性骨质疏松症治疗药物的药物经济学评价 |
| 91 | 2013 | 胥会英; 韩新民; 黄建萍 | 小儿呼吸道合胞病毒肺炎中西医对照治疗成本-效果分析 |
| 92 | 2013 | 安广文 | 三种中成药治疗稳定型心绞痛的药物经济学分析 |
| 93 | 2013 | 刘桂玲; 徐国成 | 艾迪与康莱特注射液联合化疗治疗非小细胞肺癌的成本-效果分析 |
| 94 | 2013 | 李亚玲; 李俊; 叶云; 余德智; 陈红艳 | 中西药联用治疗慢性荨麻疹临床疗效观察及其成本效果分析 |
| 95 | 2013 | 张明; 倪颖; 金伟华; 范开华 | 中西医结合治疗慢性萎缩性胃炎恢复期成本效果分析 |
| 96 | 2013 | 杨丽娟; 吴胜红; 王莉梅; 姚铭; 马明 | 急性缺血性脑卒中药物治疗的经济学评价 |
| 97 | 2013 | 王燕莹; 窦丽萍; 钟薏 | 中药制剂联合化疗治疗老年晚期非小细胞肺癌疗效及成本效果分析 |
| 98 | 2013 | 翟素红; 王中伟; 曹学东; 王美玲 | 益胃汤中药饮片煎剂与配方颗粒的成本效果分析 |
| 99 | 2013 | 王洪涛 | 疏血通注射剂治疗脑梗死的用药有效性、安全性及经济性评价 |
| 100 | 2013 | 李家伟; 张璐莹; 王峦; 杨丽; 周晔; 刘仲明; 沈堂彪; 何琦环; 程晓明 | 中医与西医门诊治疗同病种费用的经济学评价 |
| 101 | 2013 | 陈健清; 陈贵全; 简晓顺; 杨任 | 两种抗肿瘤中药注射剂在乳腺癌化疗应用的药物经济学分析 |
| 102 | 2013 | 宋建武; 李翠兵; 宋欣颖; 周晓峰 | 中药注射剂辅助PD方案治疗晚期卵巢癌的临床效果与经济学评价 |
| 103 | 2013 | 郭磊 | 两种中药注射液在冠心病心绞痛中的成本效果分析 |
| 104 | 2013 | 王萍; 王大荣; 万鸿; 朱晓东; 刘静 | 不同中成药注射液辅助治疗非小细胞肺癌的有效性与经济学评价 |
| 105 | 2014 | 张黎明 | 中西医结合治疗脑梗死及其后遗症的药物经济学分析 |
| 106 | 2014 | 季聪华; 洪雪文; 邵琼; 刘姗; 张颖 | 中医诊疗再生障碍性贫血成本-效果分析 |
| 107 | 2014 | 张香菊; 张明艳; 龚育杭 | 临床路径下急性脑梗死药物治疗方案的成本-效果分析 |
| 108 | 2014 | 赵雪梅 | 中西医结合治疗消渴(2型糖尿病)经济学评价 |
| 109 | 2014 | 夏红雷; 柳辉高 | 5种中成药治疗外感风热型慢性咽炎的药物成本-效果比较 |
| 110 | 2014 | 王好艺 | 生脉胶囊与复方丹参滴丸治疗不稳定型心绞痛的药物经济学分析 |
| 111 | 2014 | 田靖; 彭玲娜 | 两种中西医结合方案治疗老年女性下尿路感染的成本-效果分析 |
| 112 | 2014 | 吴小建 | 艾愈胶囊辅助化疗治疗非小细胞肺癌的药物经济学评价 |
| 113 | 2014 | 杨丹 | 中西医结合单元疗法对于急性冠脉综合征患者的临床成本效果分析 |
| 114 | 2014 | 聂含竹 | 中医外治综合疗法对产后缺乳的临床疗效和卫生经济学的评价 |
| 115 | 2014 | 李洪超; 许扬扬 | 芪明颗粒治疗糖尿病视网膜病变的疗效及经济性评价 |
| 116 | 2014 | 陈国新 | 痰热清与罗氏芬注射液治疗急性肺炎用药方案的费用-效果分析 |
| 117 | 2014 | 王怀冲; 徐颖颖; 张相彩; 王翠莲; 曹佳薇; 何俏军 | 中药与免疫调节剂辅助治疗复治肺结核的疗效及经济学评价 |
| 118 | 2014 | 曹晓岚; 赵世珂; 胡浩; 田立; 王白玲; 陶素爱; 陈建强; 付巍; 王金桥; 李东晓 | 急性缺血性中风病中医综合治疗方案疗效及卫生经济学评价 |
| 119 | 2014 | 赵萍; 张学会; 孟玲 | 两种中药注射剂治疗糖尿病周围神经病变的成本效果分析 |
| 120 | 2014 | 郝光磊 | 基于贝叶斯混合处理比较法对口服降糖药治疗Ⅱ型糖尿病成本—效果评价研究 |
| 121 | 2014 | 武小强 | 固肾通络胶囊治疗糖尿病肾病的临床研究及成本效果分析 |
| 122 | 2014 | 吴俊燕 | 黑地黄丸治疗慢性肾衰竭脾肾两虚兼湿浊证的成本效益分析 |
| 123 | 2014 | 石亚飞; 闫荟; 孙世光; 王瑞; 孙晓迪; 李阳; 王苏会 | 两种丹参类中药注射剂治疗冠心病心绞痛的系统评价及其药物经济学分析 |
| 124 | 2014 | 刘薇薇; 陈慧; 任明; 宫淑琴; 张同元; 王浩 | 不同治疗方案对小儿反复呼吸道感染成本-效果分析 |
| 125 | 2014 | 李筱颖; 于涛; 陈新林 | 中医药联合序贯疗法根除Hp相关性胃炎成本效用分析 |
| 126 | 2014 | 杨丽娟; 闫丽娟; 王玉英 | 中药提取物治疗急性缺血性脑梗死的成本效果分析 |
| 127 | 2015 | 李彬; 陈静; 赵慧辉; 王居新; 李婷; 王娟; 王伟 | 依据证候要素辨证治疗慢性心衰的成本-效用分析 |
| 128 | 2015 | 何宇峰; 李建婷; 杨楠; 王本国; 黄晓煌 | 中西医结合卒中单元治疗血管性痴呆的成本-效果分析 |
| 129 | 2015 | 王沛陵; 李晶莹; 付娜; 段小宛 | 两种方案治疗慢性乙型肝炎肝纤维化的成本-效果分析 |
| 130 | 2015 | 贾兴泽; 赵婷丽; 林荣; 李成田 | 中西医结合治不稳定心绞痛成本-效果分析 |
| 131 | 2015 | 胡明; 胡相冰 | 不同中成药辅助治疗急性病毒性心肌炎的成本-效果分析 |
| 132 | 2015 | 王华真; 张彦旭 | 带状疱疹中西治疗方案的药物经济学比较 |
| 133 | 2015 | 王怀冲 | 中药制剂与免疫调节剂辅助治疗复治肺结核队列研究及经济学评价 |
| 134 | 2015 | 罗骞; 涂星; 熊芬; 苏芬丽 | 醒脑静注射液联合纳洛酮治疗急性酒精中毒的经济学分析 |
| 135 | 2015 | 邹小雅; 王小玲; 郑玉容 | 几种常用中药注射剂治疗小儿外感发热药物经济学研究 |
| 136 | 2015 | 王文林; 吴敏; 赵艳花; 倪市毛 | 两种中药注射剂辅助治疗冠心病心衰的成本-效果分析 |
| 137 | 2015 | 刘海艳; 任吉祥; 王健; 张影; 吕志国; 赵建军 | 破血化瘀、填精补髓中药汤剂治疗脑出血急性期的药物经济学评价 |
| 138 | 2015 | 韦邦 | 3种中药注射液治疗急性脑梗死的成本-效果分析 |
| 139 | 2015 | 孙茂 | 基于贝叶斯方法两种药物治疗脑梗塞（后遗症期）成本效果模型构建研究 |
| 140 | 2015 | 李彬 | 基于证候要素辨证组方治疗慢性心衰的临床研究及成本—效果分析 |
| 141 | 2015 | 吴锗珊; 林淑玲; 罗钦宏; 蔡陈浩; 洪锐杰 | 中药注射剂辅助NP方案治疗老年非小细胞肺癌的循证药物经济学评价 |
| 142 | 2015 | 王慧; 李婷; 左文; 葛艳 | 3种中药注射剂治疗中风方案成本-效果分析 |
| 143 | 2015 | 魏云侠 | 联合应用破血化瘀，填精补髓法中药汤剂治疗脑出血急性期的成本效果评价 |
| 144 | 2015 | 肖凌; 周蕾; 赵丽红; 田建辉; 徐蔚杰; 刘吟絮; 朱丽华; 李和根 | 三种方案治疗Ⅲ-Ⅳ期非小细胞肺癌的成本-效益研究 |
| 145 | 2015 | 刘俊; 汪亚玲; 白劲松; 李重熙; 田波; 陈欣 | 中药、克力芝治疗HAART后免疫重建不良患者疗效及成本效益 |
| 146 | 2016 | 赵庆大; 孙利华 | 中西医结合治疗中晚期非小细胞肺癌患者的成本-效果分析 |
| 147 | 2016 | 张学斌; 徐菲; 刘国恩 | 疏血通注射液治疗缺血性脑卒中药物经济学评价 |
| 148 | 2016 | 母敏 | 3种中成药治疗肝纤维化的药物经济学评价 |
| 149 | 2016 | 刘军 | 中药注射液治疗急性脑梗死的成本-效果分析 |
| 150 | 2016 | 赖珺; 李丹; 巫莉萍; 薛小燕; 张四青 | 不同活血化瘀中药注射液对急性脑梗死的疗效及成本-效果分析 |
| 151 | 2016 | 张海明 | 中药注射剂辅助治疗非小细胞肺癌的临床疗效及经济学评价 |
| 152 | 2016 | 王丽红 | 3种中药注射剂治疗中风的临床疗效及药物经济学成本-效果分析 |
| 153 | 2016 | 张淑云; 沈翔 | 祛瘀消肿汤预防老年髋部骨折术后深静脉血栓的临床观察与药物经济学分析 |
| 154 | 2017 | 刘笃佳; 王媛媛; 张倩; 郭桐君; 张亚; 彭左旗; 裴开颜; 马旭 | 育龄人群孕前优生中医预防保健服务包的成本-效果分析 |
| 155 | 2017 | 谷红苹; 马伟明; 许瞻; 应华娜; 倪约翰 | 止嗽理肺汤治疗社区获得性肺炎药物经济学评价 |
| 156 | 2017 | 孙毅; 李慧琴 | 多中心评价银杏内酯注射液治疗缺血性脑卒中药物经济学 |
| 157 | 2017 | 竺佳 | 保妇康和燥湿清毒饮治疗宫颈炎合并HPV感染的成本及效果分析 |
| 158 | 2017 | 王海兵; 张天豪; 张学斌 | 冠心静胶囊治疗冠心病心绞痛的临床疗效及经济学评价 |
| 159 | 2017 | 李倩 | 复方黄柏液治疗疮疡阳证的临床评价及成本效果分析 |
| 160 | 2017 | 胡烨焰; 许汝言; 叶露 | 中药注射剂辅助肺腺癌一线化疗的成本效果分析 |
| 161 | 2017 | 隗秀荣 | 喜炎平注射液治疗小儿支气管炎有效性、安全性、经济性系统评价 |
| 162 | 2017 | 王婷 | 两种中药注射液治疗儿童手足口病的疗效、安全性及经济学评价 |
| 163 | 2017 | 孟欣 | 缺血性脑卒中治疗的药物经济学研究 |
| 164 | 2017 | 王振刚 | 中药汤剂联合西药治疗室性早搏的效果及对经济学指标的影响 |
| 165 | 2017 | 邓佳; 宋香清 | 2种中药注射剂治疗小儿急性上呼吸道感染的成本—效果比及社会效益评价 |
| 166 | 2018 | 李小波; 杨龙娜; 周国坚 | 三种中成药在左氧氟沙星基础上辅助治疗社区获得性肺炎(非重症,成人)的成本效果分析 |
| 167 | 2018 | 王茜 | 登革热门诊患者治疗方案的药物经济学分析 |
| 168 | 2018 | 左新河; 王文见; 刘巍巍; 蔡惠群; 汪陆玲; 王玉双; 吕俊生; 钟华; 童传明; 华川; 陈继东; 赵刚; 黄志军; 李和林 | 小金胶囊治疗甲状腺结节药物经济学研究 |
| 169 | 2018 | 季传平; 何纯; 李慧琴 | 多中心评价银杏内酯注射液治疗重症缺血性脑卒中药物经济学 |
| 170 | 2018 | 蔡秋晗; 胡思源; 刘颖; 姜翠莲; 钟成梁; 李磊; 邹婷 | 儿童清咽解热口服液治疗小儿急性咽炎(肺胃实热证)的药物经济学评价 |
| 171 | 2018 | 陈昌明; 刘杰; 张英; 林洪生 | 晚期非小细胞肺癌患者三种治疗方案经济学评价 |
| 172 | 2018 | 黄斌 | 两种不同活血化瘀中药注射液对急性脑梗死的疗效及成本-效果分析 |
| 173 | 2018 | 龚睿; 付南思 | 2种中药注射液预防骨科围手术期深静脉血栓形成的成本-效果分析 |
| 174 | 2019 | 张洪钦; 陈秋琴; 陈寿菲 | 临床药师探讨中西医结合治疗小儿喘息性支气管炎的临床疗效及药物经济学研究 |
| 175 | 2019 | 国延泉 | 炎琥宁、热毒宁治疗儿童支气管肺炎药物经济学对比研究 |
| 176 | 2019 | 许瞻; 谷红苹; 倪约翰; 应华娜 | 社区获得性肺炎(痰热壅肺证)中医临床路径的药物经济学研究 |
| 177 | 2019 | 袁静 | 三种中药制剂治疗冠心病心绞痛的成本-效果分析 |
| 178 | 2019 | 李国英; 陈良; 刘军 | 三种中成药治疗肝肾阴虚型高血压成本-效果分析 |
| 179 | 2019 | 王海霞; 吕光阳 | 常用中药贴片治疗骨科疼痛疾病的药物经济学评价 |
| 180 | 2019 | 彭艳君 | 六合汤汤剂与单味颗粒剂治疗气虚湿热型腰痛症的临床疗效及药物经济学成本-效果分析 |
| 181 | 2019 | 陈迎春 | 药物经济学评价在中药配方颗粒调剂中的应用分析 |
| 182 | 2019 | 陈迎春 | 药物经济学方法在中药饮片和中药配方颗粒剂中的评价现状观察 |
| 183 | 2019 | 金明; 吴力强 | 活血化瘀类中药配方颗粒与中药饮片的成本-效果比较研究 |
| 184 | 2019 | 朱琦敏 | 银杏酮酯滴丸、麝香保心丸及灯盏生脉胶囊治疗稳定型心绞痛药物经济学分析 |
| 185 | 2019 | 吴佳瑶; 张颖; 段蓉; 李正翔 | 安胎丸治疗复发性流产的疗效观察及药物经济学评价 |
| 186 | 2019 | 张岚; 钱夕元; 阮彤 | 基于Lasso的慢性心衰病人中西医疗效对比研究 |
| 187 | 2019 | 王紫怡; 姚仲青; 曾海松; 宋敏; 丁选胜 | 柴芩清宁胶囊与清开灵胶囊治疗急性上呼吸道感染的成本-效果分析 |
| 188 | 2019 | 吕瑞民; 王英臣 | 养心氏片治疗气虚血瘀型心绞痛的疗效与成本-效果分析 |
| 189 | 2019 | 邓治国; 唐芙蓉 | 3种中药注射剂辅助治疗糖尿病周围神经病变的有效性与经济学评价 |
| 190 | 2020 | 胡静; 徐嘉路; 韩漫漫; 董祺; 杨骏; 缪红; 徐小芳 | 活血化瘀类中药注射剂在颈椎病非手术治疗中的药物经济学评价分析 |
| 191 | 2020 | 徐焕翔; 蔡永进 | 两种中药配方颗粒调剂的药物经济学评价 |
| 192 | 2020 | 李梦岚; 周长凤; 于蕾 | 银杏叶提取物注射液、注射用血栓通和丹红注射液治疗脑梗死的成本-效果分析 |
| 193 | 2020 | 吕小琴 | 三种中成药治疗方案用于寻常性痤疮的成本效果分析 |

**Table S3. Included English Studies**

| **Number** | **Year** | **Author** | **Title** |
| --- | --- | --- | --- |
| 1 | 2006 | Ratcliffe, J; Thomas, K J; MacPherson, H; Brazier, J | A randomised controlled trial of acupuncture care for persistent low back pain: cost effectiveness analysis |
| 2 | 2012 | Deng, Z Q; Zheng, H; Zhao, L; Zhou, S Y; Li, Y; Liang, F R | Health economic evaluation of acupuncture along meridians for treating migraine in China: results from a randomized controlled trial |
| 3 | 2015 | Sun, X; Guo, L; Shang, H; Ren, M; Wang, Y; Huo, D; Lei, X; Wang, H; Zhai, J | The cost-effectiveness analysis of JinQi Jiangtang tablets for the treatment on prediabetes: a randomized, double-blind, placebo-controlled, multicenter design |
| 4 | 2018 | Pokladnikova, J; Maresova, P; Dolejs, J; Park, A L; Wang, B; Guan, X; Musil, F | Economic analysis of acupuncture for migraine prophylaxis |
| 5 | 2018 | Dong, P; Hu, H; Guan, X; Ung, COL; Shi, L; Han, S; Yu, S | Cost-consequence analysis of salvianolate injection for the treatment of coronary heart disease |
| 6 | 2018 | Xuan, J; Huang, M; Lu, Y; Tao, L | Economic Evaluation of Safflower Yellow Injection for the Treatment of Patients with Stable Angina Pectoris in China: A Cost-Effectiveness Analysis |
| 7 | 2018 | Pach, D; Piper, M; Lotz, F; Reinhold, T; Dombrowski, M; Chang, Y; Liu, B; Bl?dt, S; Rotter, G; Icke, K; Witt, C M | Effectiveness and Cost-Effectiveness of Tuina for Chronic Neck Pain: A Randomized Controlled Trial Comparing Tuina with a No-Intervention Waiting List |
| 8 | 2014 | Li, Y; Xi, H X; Zhu, S; Yu, N; Wang, J; Li, Y; Yu, G P; Ma, X M; Zhang, J; Zhao, L P | Cost-effectiveness analysis of combined Chinese medicine and Western medicine for ischemic stroke patients |
